# The association between diabetes and head and neck squamous cell carcinoma: evidence from clinical cohort and bioinformatics analyses

**DOI:** 10.3389/fgene.2025.1660012

**Published:** 2025-08-13

**Authors:** Yurong He, Jiaming Chen, Boxuan Han, Yanming Zhao, Lizhen Hou, Jugao Fang, Meng Lian

**Affiliations:** ^1^ Department of Otorhinolaryngology Head and Neck Surgery, Beijing Tongren Hospital, Capital Medical University, Beijing, China; ^2^ Key Laboratory of Otorhinolaryngology Head and Neck Surgery (Capital Medical University), Ministry of Education, Beijing, China; ^3^ The 2nd Department of Head and Neck Surgery, Department of Oncoplastic Surgery, Hunan Cancer Hospital and the Affiliated Cancer Hospital of Xiangya School of Medicine, Central South University, Changsha, China

**Keywords:** diabetes mellitus, head and neck squamous cell carcinoma, comorbidity, gene overlap, bioinformatics, co-occurrence modeling, sex differences

## Abstract

**Introduction:**

Diabetes mellitus (DM) is a known risk factor for various cancers, but its relationship with head and neck squamous cell carcinoma (HNSCC) remains unclear. This study explores clinical and molecular links between DM and HNSCC through integrative analyses of patient data and bioinformatics.

**Methods:**

A retrospective cohort of 728 HNSCC patients was analyzed to assess sex-specific co-occurrence with DM. A simulation-based epidemiological model quantified associations based on observed clinical data and population incidence rates. Literature-based data mining was used to extract gene–disease associations for DM and HNSCC, followed by functional enrichment, pathway and network analyses of overlapping genes.

**Results:**

The simulation revealed a significant association between DM and HNSCC, stronger in males (Odds Ratio [OR] = 3.03, p = 6.28 × 10^−50^) than in females (OR = 2.18, p = 8.7 × 10^−12^). Data mining uncovered 3,489 overlapping genes (OR = 6.73, p < 4.95 × 10^−319^), including nine key genes (GPX4, NLRP3, CASP3, HOTAIR, SRC, IGF2BP2, APP, CYP2C19, and PVT1) tightly interconnected and functionally enriched in inflammation, metabolism, and neurological signaling pathways. Four genes—CYP2C19, NLRP3, PVT1, and APP—appear central to DM’s influence on HNSCC via the protein–protein interaction (PPI) network.

**Conclusion:**

These findings reveal a significant clinical and molecular connection between DM and HNSCC, especially in males, and highlight potential targets for future prevention and treatment strategies.

## 1 Introduction

Diabetes mellitus (DM) is a chronic metabolic disorder characterized by elevated blood glucose levels due to insufficient insulin production or ineffective insulin utilization. Globally, approximately 537 million adults were living with DM in 2021—representing an overall population prevalence of approximately 0.069 (or 6.9%)—and this number is projected to rise to 643 million by 2030 (Hossain, et al., 2024). Consider the overall Type 2 DM—the predominant form—is strongly linked to recognized risk factors, including obesity, sedentary behavior, unhealthy dietary habits, and genetic predisposition (Hossain, et al., 2024; Ismail, et al., 2021). These contributors fuel a growing global health burden and underscore the importance of effective management and prevention strategies.

Head and neck squamous cell carcinoma (HNSCC) refers to malignant tumors originating from the mucosal linings of the oral cavity, pharynx, and larynx. In the United States, HNSCC accounts for approximately 3% of all cancers, corresponding to an estimated 54,540 new cases and 11,580 deaths in 2023 (Barsouk, et al., 2023). The incidence is significantly higher in males than females, with a ratio of about 2:1 (incidence rates: 0.0152% in males *versus* 0.00761% in females), and is strongly linked to modifiable risk factors such as tobacco use and heavy alcohol consumption (Barsouk, et al., 2023). Furthermore, chronic infection with high-risk human papillomavirus (HPV-16) is a critical etiologic factor in the rising incidence of oropharyngeal carcinoma (Galati, et al., 2022).

Existing research indicates that DM may influence cancer risk through mechanisms such as insulin resistance and chronic inflammation, which could potentially affect the development of various malignancies. Obesity, a major risk factor for type 2 DM, is also independently associated with increased cancer risk, highlighting a complex interplay among chronic conditions (Christensen and Nelson, 2025). While DM is a recognized risk factor for several cancers—including liver and pancreatic malignancies—its direct association with HNSCC remains less clearly defined (Chen, et al., 2025a; Moreland, et al., 2025). Recent studies in malignancies treated with immunotherapy, such as metastatic melanoma, suggest that DM may influence tumor biology, biomarker expression, and therapeutic response through immune checkpoint modulation (Cortellini, et al., 2023; Mallardo, et al., 2022; Mallardo, et al., 2023). These findings are particularly relevant given the expanding use of immunotherapy in HNSCC and underscore the need to explore DM’s role in tumor microenvironment and treatment outcomes.

Recent clinical and meta-analytic evidence has further supported the association between DM and HNSCC. Mirestean et al. (2023) analyzed real-world data and observed that diabetes may influence the clinical profile of patients with head and neck cancer, reinforcing the clinical significance of this comorbidity (Mirestean, et al., 2023). In a comprehensive meta-analysis, Yan et al. (2021) reported that type 2 diabetes mellitus is associated with an increased risk across several HNSCC subtypes, emphasizing the need for mechanistic investigations into this relationship (Yan, et al., 2021).

Emerging evidence suggests that DM may exacerbate clinical complications in patients with HNSCC. For example, individuals with both conditions have shown an increased risk of wound debridement and thyroid dysfunction during radiotherapy (Fiore, et al., 2025; Zhang at al., 2025a). Moreover, DM may influence pain thresholds in cancer patients and alter therapeutic responses, potentially impacting overall cancer outcomes (Zhu, et al., 2025). Interestingly, some studies have proposed a potential protective effect of certain DM treatments against cancer development, underscoring the complexity of DM–cancer comorbidity (Yen, et al., 2025).

Despite these findings, no direct causal link between DM and increased incidence of HNSCC has been established. This lack of definitive evidence highlights the urgent need for more rigorous research to understand whether, and how, DM may contribute to the pathogenesis or progression of HNSCC.

This study aims to elucidate the relationship between DM and HNSCC by integrating clinical cohort analysis with bioinformatics approaches. By identifying potential biological mechanisms and shared risk factors, the study seeks to fill critical knowledge gaps regarding the comorbidity of these two diseases. We hypothesize that DM may influence the development or progression of HNSCC through overlapping molecular pathways or systemic risk factors. The insights gained from this research may contribute to improved prevention strategies, therapeutic interventions, and overall patient management.

## 2 Methods

### 2.1 Study workflow

This study employed an integrative workflow combining clinical data analysis, simulation-based epidemiological modeling, and bioinformatics approaches to explore the association and potential molecular links between DM and HNSCC. First, a retrospective clinical cohort of 728 HNSCC patients was analyzed to assess the sex-specific co-occurrence of DM and HNSCC. A simulation-based framework was then applied to quantify the sex-stratified association between the two diseases using population incidence rates and observed clinical data. To identify shared molecular mechanisms, large-scale literature data mining was conducted to extract gene–disease associations for both DM and HNSCC across 19,924 human genes. Overlapping genes were identified and subjected to functional enrichment analysis and protein–protein interaction (PPI) network construction. Finally, a directed gene network was built to map potential biological pathways linking DM and HNSCC, highlighting candidate mediators of cross-disease effects.

### 2.2 Patient selection and clinical data

A total of 728 patients diagnosed with HNSCC who received treatment at the Department of Head and Neck Surgery, Beijing Tongren Hospital, between January 2023 and August 2024 were retrospectively enrolled in this study. The cohort exhibited a male predominance (692 males vs. 36 females), with a median age of 61 years (range: 14–91 years). Clinical parameters, including age, sex, TNM stage, and DM history, were obtained from medical records. The study was approved by the Ethics Committee of Beijing Tongren Hospital (approval number: TREC2024-KYS291) and conducted in accordance with relevant ethical guidelines. Informed consent was waived due to the retrospective study design. Table 1 present the baseline characteristics of patients.

**TABLE 1:** Baseline characteristics of patients with head and neck squamous cell carcinoma (HNSCC).

Variable	Statistic/Count	Variable	Statistic/Count
Sex	Male: 858, Female: 36	T_stage	1: 191, 2: 194, 3: 239, 4: 270
Age (years)	Mean: 61.1, Std Dev: 10.4	N_stage	0: 449, 1: 212, 2: 152, 3: 46, 4: 35
DM	No: 805, Yes: 89	M_stage	M0 (0): 893, M1 (1): 1

A detailed list of clinical data is provided in Supplementary Table S1, which also includes the HNSCC subtypes (column: “Cancer Subtypes”). However, due to limited sample sizes within certain subgroups, subgroup analyses by tumor subtype were not performed.

### 2.3 Co-occurrence modeling of DM and HNSCC by sex

We performed co-occurrence modeling to quantify the association between DM and head and neck squamous cell carcinoma (HNSCC), stratified by sex. A simulation-based framework was used to integrate observed co-occurrence data with sex-specific population incidence rates. This modeling approach served to standardize and scale the observed clinical co-occurrence rates to a representative population level, allowing for sex-specific quantification of risk (e.g., odds ratios and relative risks) under epidemiologically realistic assumptions. It enhances interpretability, generalizability, and reproducibility by bridging real-world clinical observations with broader population data.

#### 2.3.1 Estimation of sex-stratified contingency tables

Separate simulations were conducted for male and female populations, each consisting of 10,000 individuals. The prevalence of DM was assumed to be equal across sexes, while HNSCC incidence rates were modeled using sex-specific estimates from population data. Observed co-occurrence rates from our study cohort were used to inform the conditional probability of HNSCC given DM for each sex.

The inputs for the simulation model were empirically derived. The sex-specific conditional probabilities of HNSCC among diabetic patients were calculated based on our observed clinical cohort, comprising 728 patients. For males, this probability was calculated from 127 HNSCC cases among 692 diabetic males, yielding P(HNSCC | DM) = 0.1835. For females, it was calculated from 5 HNSCC cases among 36 diabetic females, yielding P(HNSCC | DM) = 0.1389. These empirically derived probabilities were integrated with nationally reported disease prevalence/incidence rates (Barsouk, et al., 2023; Hossain, et al., 2024) to simulate a representative population of 10 million individuals per sex, generating contingency tables for co-occurrence analysis. This ensured that the simulation was rooted in both real-world data and population-level statistics.

#### 2.3.2 The modeling process comprised the following steps

1) The conditional probability of HNSCC given DM, $P\left(HNSCC|DM\right)$ , was estimated from the observed data for each sex.
2) The joint probability of co-occurrence, $P\left(HNSCC\cap DM\right)$ , was computed using Bayes’ theorem:

$$P\left(HNSCC\cap DM\right)=P\left(DM\right)\times P\left(HNSCC|DM\right)$$

3) Based on this joint probability and population incidence rates, we estimated the expected number of individuals in each cell of a 2 × 2 contingency table for a simulated population of 10,000 individuals:

**Table:** 

Fisher-exact test	HNSCC	No HNSCC
DM	a (DM and HNSCC)	b (DM and No HNSCC)
No DM	c (No DM and HNSCC)	d (No DM and No HNSCC)

Where: a = number of individuals with both DM and HNSCC; b = number of individuals with DM but without HNSCC; c = number of individuals without DM but with HNSCC; d = number of individuals without either condition.

This simulation process was applied separately to male and female populations to generate sex-stratified contingency tables.

#### 2.3.3 Statistical analysis of Co-occurrence

For each sex-specific contingency table, we calculated standard measures of association to quantify the relationship between DM and HNSCC.

• Odds Ratio (OR):

$$OR=\frac{a/b}{c/d}=\frac{ad}{bc}$$

Representing the odds of HNSCC among individuals with DM relative to those without.

• Relative Risk (RR):

$$RR=\frac{a/\left(a+b\right)}{c/\left(c+d\right)}$$

Representing the probability (risk) of HNSCC in individuals with DM compared to those without.

• Fisher’s Exact Test:

Fisher’s Exact Test was performed on each sex-specific contingency table to evaluate whether the observed co-occurrence of DM and HNSCC differed significantly from what would be expected under the null hypothesis of independence.

This modeling framework enabled a transparent and reproducible quantification of the sex-specific association between DM and HNSCC in the simulated populations.

### 2.4 Literature data mining for gene–disease associations

To identify genes potentially associated with DM and head and neck squamous cell carcinoma (HNSCC), we performed large-scale literature data mining covering 19,924 human genes. Two primary tools were used for literature retrieval: the Entrez API (https://www.ncbi.nlm.nih.gov/Entrez/), which provides automated access to biomedical literature in PubMed, and the AIC Bioinformatics Toolbox (ABT; https://www.gousinfo.com/en/userguide.html), an AI-powered platform that extracts gene–disease relationships from both a proprietary literature database (ABD) and public sources such as PubMed.

For each gene, retrieved information—including article titles, publication dates, PubMed IDs (PMIDs), Digital Object Identifiers (DOIs), and abstracts—was compiled into a structured Excel file for downstream analysis. The combined use of these tools enabled large-scale, efficient extraction of relevant literature, supporting the identification of candidate genes based on published evidence.

An Adjusted Binomial Method (ABM) (Lian, et al., 2025) was employed to assess the reliability of each gene–disease association identified through literature data mining (LDM). To control for multiple comparisons, we applied a False Discovery Rate (FDR) correction, retaining only associations with an FDR-adjusted p-value ≤0.01. This filtering ensured that subsequent analyses focused on statistically significant and biologically plausible gene–disease relationships (Lian, et al., 2025).

The statistical significance of gene–disease associations was computed using the following formula:

$$p-value=P\left(X\geq n_{p}\right)=binom.sf\left(n_{p}-1,N,{\;p}_{0}\right)$$

where binom.sf is the survival function of the binomial distribution, n_p is the observed number of positive polarity findings, N is the total number of polarity-adjusted observations, and p_0_ is the expected null proportion under random association.

### 2.5 Cross-disease gene overlap analysis

To identify potential shared molecular mechanisms between DM and HNSCC, we compared gene lists to evaluate the statistical significance of the observed overlap. The intersection was visualized using a Venn diagram. Comparisons were conducted using both the complete set of disease-associated genes and a subset of significant genes, with subsequent functional and network analyses focused primarily on this prioritized gene set.associated with each disease to identify both unique and overlapping genes. Fisher’s exact test was applied.

### 2.6 Functional analysis of overlapping genes

To better understand the shared biological basis of DM and HNSCC, we performed functional annotation and protein–protein interaction (PPI) network analysis on the overlapping genes.

For functional annotation, we used the DAVID database (https://david.ncifcrf.gov) to identify enriched biological processes, cellular components, molecular functions (GO terms: GOTERM_BP_DIRECT, GOTERM_CC_DIRECT, and GOTERM_MF_DIRECT), and known pathways (BBID, BIOCARTA, and KEGG_PATHWAY). This analysis provided insights into the biological roles and pathways associated with the overlapping genes.

For PPI network analysis, we constructed a network using experimentally validated and literature-supported interactions. The network structure was evaluated using key topological measures, including network density, average path length, clustering coefficient, diameter, and centrality metrics. Genes that ranked highly across centrality measures were designated as hub genes, which may play important roles in the shared pathophysiology of DM and HNSCC. Functional enrichment analysis of hub genes was also performed.

### 2.7 Construction of directed pathways connecting DM and HNSCC

To explore potential biological pathways linking DM and HNSCC, we constructed a directed gene network based on overlapping genes with statistically significant associations to both diseases.

Known or inferred gene–gene interactions were integrated to form directional paths (e.g., DM → Gene A → HNSCC), representing possible biological routes through which one disease may influence the other. This network highlights candidate causal pathways and hub genes that may mediate cross-disease effects, providing a foundation for future mechanistic studies.

## 3 Results

### 3.1 Co-occurrence modeling of diabetes and HNSCC by sex

We conducted sex-stratified co-occurrence modeling of Diabetes and Head and Neck Squamous Cell Carcinoma (HNSCC) using a simulation-based approach with a hypothetical population of 10,000,000 individuals for each sex. The prevalence of Diabetes was set at 6.9% for both sexes, based on epidemiological estimates. The incidence of HNSCC was modeled using sex-specific values: 0.000152 for males and 0.0000761 for females.

In our observed cohort, the conditional probability of HNSCC given Diabetes was estimated as P(HNSCC∣Diabetes) = 0.1835 for males (127 cases of HNSCC among 692 diabetic males) and P(HNSCC∣Diabetes) = 0.1389 for females (5 cases among 36 diabetic females). Using these values and the population-based disease rates, we simulated contingency tables for each sex.

For males, the co-occurrence modeling estimated 279 individuals with both Diabetes and HNSCC, 689,721 with Diabetes only, 1,241 with HNSCC only, and 9,308,759 with neither condition. For females, the simulation estimated 106 individuals with both conditions, 689,894 with Diabetes only, 655 with HNSCC only, and 9,309,345 with neither.

Statistical analysis of these contingency tables showed a stronger association between Diabetes and HNSCC in males. Among males, the Odds Ratio (OR) and Relative Risk (RR) were both estimated at 3.03, with a highly significant Fisher’s Exact Test p-value of 6.28 × 10^−50^. Among females, both OR and RR were estimated at 2.18, with a p-value of 8.7 × 10^−12^. The details can be found in Table 2.

**TABLE 2:** Co-occurrence modeling results for diabetes and HNSCC, stratified by sex.

Sex	Diabetes and HNSCC (a)	Diabetes only (b)	HNSCC only (c)	Neither (d)	Odds ratio (OR)	Relative risk (RR)	p-value
Male	279	689,721	1,241	9,308,759	3.03	3.03	6.28 × 10^−50^
Female	106	689,894	655	9,309,345	2.18	2.18	8.7 × 10^−12^

Note: a–d represent estimated counts in the simulated population. OR, odds ratio; RR, Relative Risk. Simulations were based on observed conditional probabilities and sex-specific population incidence rates of diabetes and HNSCC.

These findings indicate a statistically significant positive association between Diabetes and HNSCC in both sexes, with the magnitude of association notably stronger in males.

### 3.2 Disease-gene identification and comparison results

**FIGURE 1:**
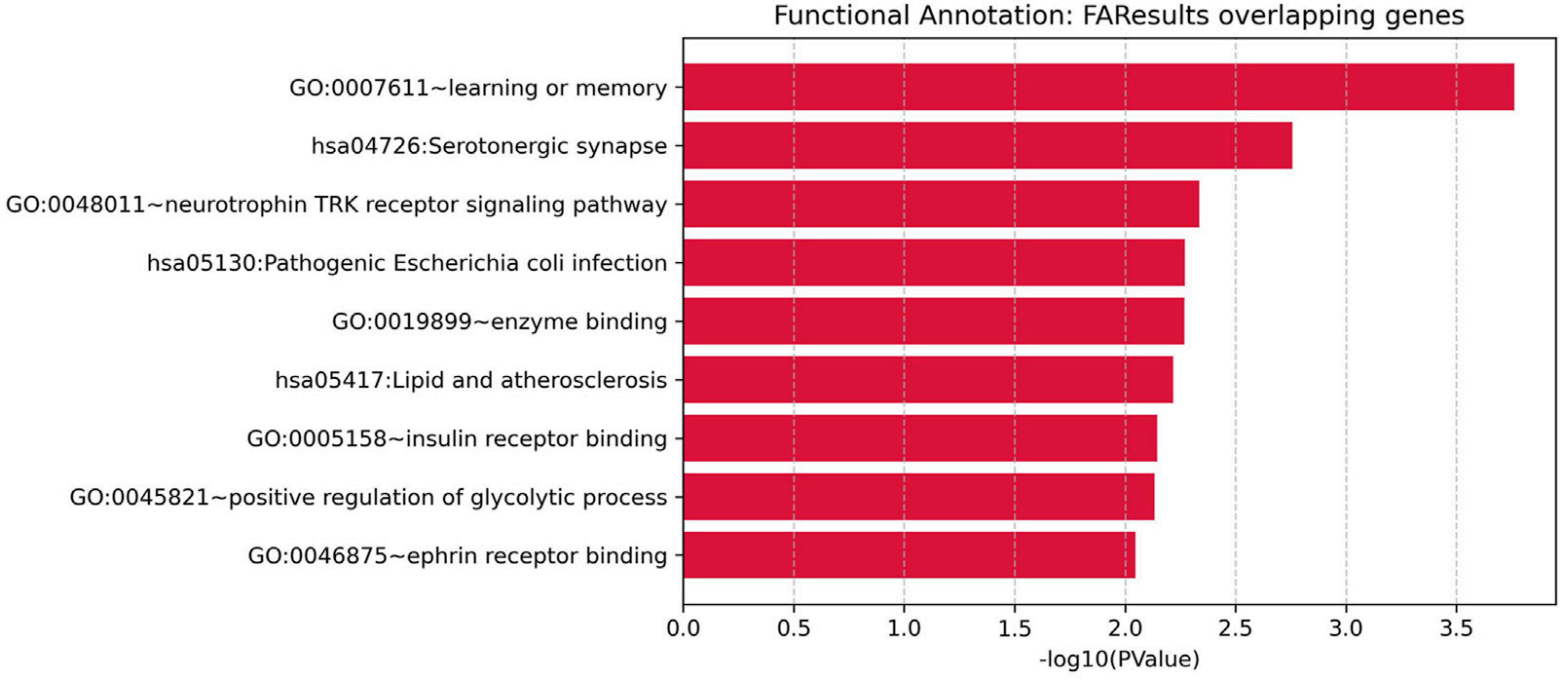
Venn diagrams illustrating the overlap between genes associated with Diabetes and Head and Neck Squamous Cell Carcinoma (HNSCC).

Out of a total of 19,924 genes analyzed, the AI-driven literature mining identified 7,403 genes linked to Diabetes mellitus, supported by 25,277 references, and 4,954 genes linked to Head and Neck Squamous Cell Carcinoma (HNSCC), supported by 14,331 references. A total of 3,489 genes were found to be common to both conditions. This overlap is highly significant, with a Fisher’s exact test showing an odds ratio (OR) of 6.73 and a p-value below 4.95 × 10^−319^, as presented in Table 3 and Figure 1, indicating a strong enrichment of shared genes between Diabetes and HNSCC. When a more rigorous criterion was applied, limiting associations to those with an FDR-adjusted q-value of 0.01 or less, 195 Diabetes-associated genes and 124 HNSCC-associated genes met this threshold, among which 9 genes overlapped significantly (OR = 8.25, p-value = 3.69 × 10^−6^). These findings provide further evidence for a meaningful genetic connection between Diabetes and HNSCC (Table 3; Figure 1).

**TABLE 3:** Overlapping genes between diabetes mellitus (DM) and HNSCC from literature-based gene association analysis.

Gene Category	Source Disease	Target disease	#Genes source	#Genes target	Overlap	Odds ratio	p-value
All genes	DM	HNSCC	7403	4954	3489	6.73	<4.95E-319
Significant Genes (p-value ≤ 0.01)	DM	HNSCC	195	124	9	8.25	3.69E-06

Note: Gene categories include all literature-identified genes and a subset meeting the significance threshold (FDR-adjusted p-value ≤0.01). Overlap significance was calculated using Fisher’s exact test.

The 9 significant overlapping genes identified for further analysis are: GPX4, NLRP3, CASP3, HOTAIR, SRC, IGF2BP2, APP, CYP2C19, and PVT1, as detailed in the subsequent sections.

### 3.3 PPI analysis

**FIGURE 2:**
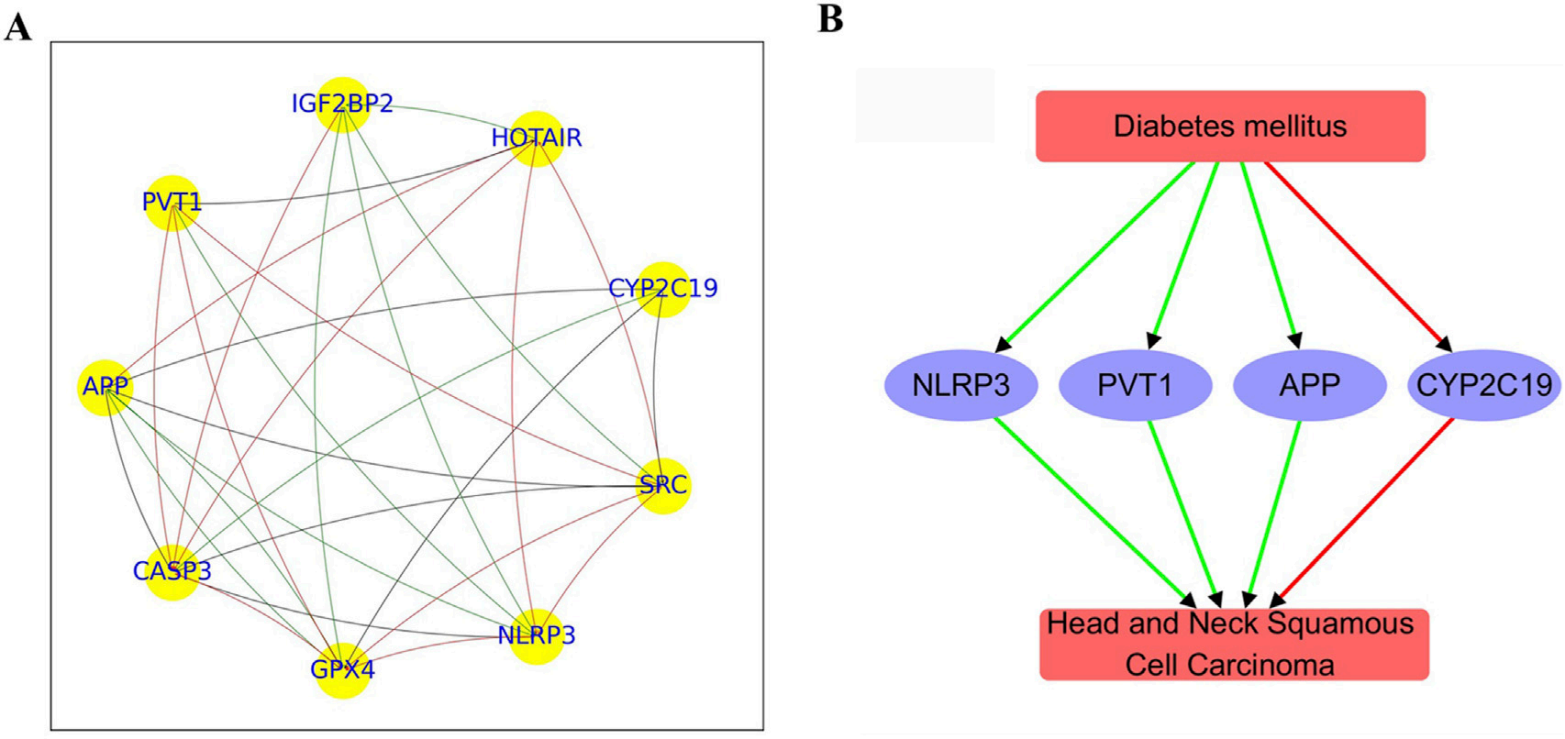
PPI network and functional pathway constructed using overlapping genes between diabetes mellitus and HNSCC. **(A)** PPI network comprising 9 genes. **(B)** Pathway illustrating connections between diabetes mellitus and HNSCC. Green edges represent positive associations; red edges indicate negative associations.

The PPI network constructed from 9 genes consisted of 29 edges, revealing a moderately dense and cohesive structure (Figure 2A). The network density was 0.40, with an average path length of 2.0 and a high clustering coefficient of 0.82, indicating substantial interconnectivity within a single connected component. The network diameter was 2, reflecting a compact topology among the nodes.

Centrality metrics identified key hub genes with important roles in the network. CASP3 exhibited the highest in-degree centrality (0.88), marking it as a major interaction target, followed by GPX4 and NLRP3 (both 0.75). Out-degree centrality was highest for PVT1 (0.62), with APP, SRC, CYP2C19, HOTAIR, and IGF2BP2 all showing moderate regulatory influence (0.5). Betweenness centrality highlighted GPX4 and SRC (0.20) alongside APP (0.19) as potential mediators of network signaling. Eigenvector centrality further underscored CASP3, SRC, NLRP3, and GPX4 (≥0.36) as influential nodes connected to other highly connected genes.

Together, these results suggest CASP3, GPX4, APP, and SRC as core hub genes, likely forming the functional core of this network module. Further functional and pathway enrichment analyses of these genes are discussed below.

### 3.4 Directed pathway connecting diabetes mellitus and HNSCC

Functional pathway analysis revealed a bidirectional regulatory relationship between Diabetes mellitus and Head and Neck Squamous Cell Carcinoma (HNSCC) mediated through a shared set of functionally relevant genes (Figure 2B). Diabetes positively regulates APP (4 references; q = 0.00015), NLRP3 (19 references; q = 0.00168), and PVT1 (4 references; q = 0.00755), while negatively regulating CYP2C19 (14 references; q = 0.00372), suggesting its influence on pathways related to inflammation, immune response, and metabolism.

These genes are also significantly altered in HNSCC. APP (4 references; q = 0.00832), NLRP3 (5 references; q = 0.00229), and PVT1 (8 references; q = 0.00229) are positively regulated in HNSCC, whereas CYP2C19 is negatively regulated (4 references; q = 0.00832). This pattern indicates that both Diabetes and HNSCC impact a common gene network involved in tumor microenvironment remodeling, inflammation, and metabolic dysregulation.

The consistent directionality and statistical significance observed in the regulation of APP, NLRP3, PVT1, and CYP2C19 suggest the possibility of a Diabetes → gene → HNSCC pathway, in which diabetes-related gene alterations may contribute to HNSCC pathogenesis. These findings highlight potential shared molecular mechanisms linking the two diseases. However, this model should be considered hypothesis-generating and does not constitute definitive evidence of causality.

### 3.5 Functional annotation analysis results

**FIGURE 3:**
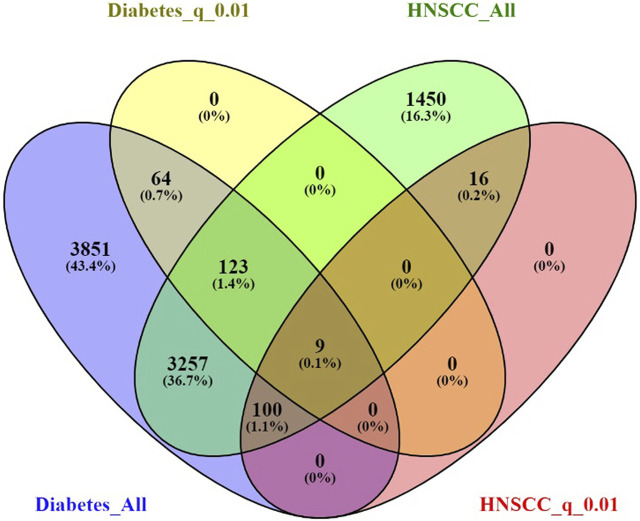
Functional enrichment analysis for overlapping genes associated with both Diabetes and Head and Neck Squamous Cell Carcinoma (HNSCC).

To explore the biological functions of the 9 overlapping genes between diabetes mellitus and HNSCC, we performed functional enrichment analysis (as described in the Methods). This analysis identified nine significantly enriched pathways or functional categories (Figure 3), primarily related to neurological processes, immune response, metabolic regulation, and atherosclerosis-related pathways.

Significant terms included “learning or memory” (GO:0007611, p = 1.73E-04), “Serotonergic synapse” (KEGG hsa04726, p = 0.00175), “neurotrophin TRK receptor signaling pathway” (GO:0048011, p = 0.00461), “Pathogenic *Escherichia coli* infection” (KEGG hsa05130, p = 0.00537), “enzyme binding” (GO:0019899, p = 0.00539), “lipid and atherosclerosis” (KEGG hsa05417, p = 0.00606), “insulin receptor binding” (GO:0005158, p = 0.00715), “positive regulation of glycolytic process” (GO:0045821, p = 0.00737), and “ephrin receptor binding” (GO:0046875, p = 0.00900).

Notably, several genes—such as APP, SRC, and CASP3—participated in multiple enriched terms, underscoring their potential roles in the shared pathogenic mechanisms linking diabetes mellitus and HNSCC, particularly through modulation of neurological signaling, immune pathways, and metabolic homeostasis.

## 4 Discussion

This study explored the clinical and molecular associations between diabetes mellitus (DM) and head and neck squamous cell carcinoma (HNSCC) through sex-stratified co-occurrence modeling, literature-based gene overlap analysis, and pathway/network analyses. The findings suggest that DM and HNSCC share significant clinical and molecular connections, with potential implications for disease monitoring and therapeutic strategies.

### 4.1 Sex-stratified Co-occurrence of diabetes and HNSCC

A significant positive association between DM and HNSCC was observed in both sexes, with a notably stronger correlation in males (OR = 3.03; RR = 3.03; p = 6.28 × 10^−50^) than in females (OR = 2.18; RR = 2.18; p = 8.7 × 10^−12^).This aligns with previous findings identifying DM as a prevalent comorbidity in laryngeal cancer patients (Repassy, et al., 2025) and a risk factor for thyroid dysfunction among HNSCC patients (Fiore, et al., 2025). Although some prior studies reported no sex differences in the co-occurrence of these diseases, our findings underscore the importance of sex-specific factors, potentially influenced by genetics, hormones, and lifestyle. The use of simulation-based modeling adds robustness to these results and emphasizes the need for tailored clinical surveillance, especially in diabetic males.

### 4.2 Shared genetic basis between diabetes and HNSCC

Gene overlap analysis revealed a significant enrichment of genes implicated in both DM and HNSCC (OR = 6.73 for all genes; OR = 8.25 for significant genes; both *p* < 10^−6^), suggesting a shared genetic and molecular foundation. While earlier studies noted metabolic dysfunction in cancer progression, they often lacked the scale and statistical rigor of our approach, which combined AI-assisted literature mining with Fisher’s exact test. This methodology enabled a comprehensive and validated identification of shared genetic markers, expanding on previous research (Repassy, et al., 2025).

However, discrepancies with other studies—such as those reporting inverse relationships between DM and cardiovascular disease (Banerjee, et al., 2025)—highlight the complexity and disease-specific nature of gene-disease interactions. The current findings support the need for further mechanistic studies to clarify how shared genes contribute to comorbidity risk across different diseases.

### 4.3 Molecular mechanisms linking diabetes and HNSCC

Functional pathway and network analyses highlight a directed regulatory relationship between DM and HNSCC, mediated by several key genes: CYP2C19, NLRP3, PVT1, and APP.

CYP2C19 is a key enzyme in the cytochrome P450 family responsible for the metabolism of numerous endogenous substances and drugs. In diabetes, hyperglycemia and chronic inflammation contribute to significantly reduced CYP2C19 enzymatic activity and protein expression (Kvitne, et al., 2024; Li et al., 2024a). This downregulation impairs hepatic detoxification processes, alters pharmacokinetics, and exacerbates oxidative stress (Chen, et al., 2024; Tian, et al., 2025). In HNSCC, genetic polymorphisms in CYP2C19 (e.g., CYP2C192) have been associated with a markedly increased risk of cancer development (OR = 3.36) and reduced therapeutic efficacy of chemotherapeutic agents like cisplatin and 5-fluorouracil, due to altered drug metabolism (Rawal, et al., 2015; Yadav, et al., 2008). These findings suggest that diabetes-induced CYP2C19 suppression may potentiate carcinogenic processes in HNSCC through impaired xenobiotic clearance and disrupted metabolic regulation.

The NLRP3 inflammasome plays a central role in the innate immune response by sensing cellular stress signals and triggering the release of pro-inflammatory cytokines like IL-1β and IL-18. In diabetes, persistent hyperglycemia and metabolic dysregulation lead to chronic NLRP3 activation in pancreatic β-cells, adipose tissue, and vasculature, thereby fueling systemic inflammation and insulin resistance (Chen S. et al., 2025; Delalat, et al., 2025). In HNSCC, particularly in oral squamous cell carcinoma (OSCC), NLRP3 contributes to cancer cell proliferation, epithelial–mesenchymal transition (EMT), and enhanced metastatic potential by activating the NF-κB and STAT3 signaling pathways (Shi, et al., 2025; Zhao, et al., 2025). This dual pro-inflammatory and pro-tumorigenic role positions NLRP3 as a critical molecular node connecting the chronic inflammatory environment of diabetes with tumorigenesis in HNSCC.

PVT1 is a long non-coding RNA that regulates gene expression through chromatin remodeling, miRNA sponging, and modulation of transcription factors. In diabetes, PVT1 is overexpressed in tissues affected by diabetic nephropathy, retinopathy, and cardiomyopathy, where it promotes fibrosis, oxidative stress, and endothelial dysfunction (Hussein, 2023; Lv, et al., 2024). Mechanistically, it acts through pathways such as TGF-β1/Smad and miR-200 family inhibition. In HNSCC, PVT1 functions as an oncogene by stabilizing MYC, suppressing tumor suppressors (e.g., p21, PTEN), and facilitating cell cycle progression, angiogenesis, and resistance to radiotherapy and chemotherapy (Li et al., 2024b; Zhao, et al., 2024). The overlapping pathogenic roles of PVT1 in tissue remodeling and malignant transformation support its role in a shared DM → PVT1 → HNSCC axis.

APP (Amyloid Precursor Protein) is a transmembrane glycoprotein best known for its role in Alzheimer’s disease, but it also regulates cell growth, survival, and adhesion. In diabetes, APP expression is upregulated in vascular tissues, liver, and kidney under hyperglycemic conditions, contributing to cellular stress, mitochondrial dysfunction, and β-cell apoptosis (Lai, et al., 2025; Zhang at al., 2025b). This may reflect a maladaptive response to metabolic overload. In HNSCC, APP has emerged as a prognostic biomarker, where its overexpression correlates with poor overall survival, increased proliferation, and invasion through the PI3K/AKT and Notch signaling pathways (Galvao, et al., 2023; Wang, et al., 2024). While a direct causal link between diabetes-induced APP dysregulation and HNSCC remains to be elucidated, the convergence of metabolic and oncogenic functions suggests a plausible mechanistic bridge.

It is important to note that while the directional relationships we proposed (e.g., DM → gene → HNSCC) are biologically plausible and supported by literature-based associations, they remain hypothesis-generating and do not establish causality. Longitudinal studies and functional validation are needed to confirm the regulatory roles of these genes in mediating cross-disease effects.

### 4.4 Converging biological and lifestyle factors in the DM–HNSCC axis

Protein-protein interaction (PPI) network analysis further revealed interconnected pathways involving inflammation, immune response, and metabolism—mechanisms common to both diseases. For instance, insulin resistance and chronic inflammation, hallmarks of DM, also contribute to HNSCC pathogenesis. Additionally, lifestyle risk factors such as smoking and alcohol consumption—common in HNSCC—can exacerbate DM by promoting oxidative stress and systemic inflammation (You, et al., 2025). Obesity, another frequent comorbidity in DM, has also been associated with worse outcomes in HNSCC (Knoedler, et al., 2025).

The functional annotation analysis of the nine overlapping genes revealed significant enrichment in pathways spanning neurological processes, immune regulation, metabolic control, and cardiovascular-related mechanisms, suggesting shared systemic dysregulation in both diabetes and HNSCC. For example, enrichment in “learning or memory” and the “neurotrophin TRK receptor signaling pathway” implicates neurotrophic support and neuronal signaling, potentially reflecting diabetic neuropathy and neuroplastic changes also relevant in cancer progression (Jin, et al., 2025). The involvement of “Serotonergic synapse” pathways may further indicate dysregulation in neurotransmitter signaling, which has been increasingly linked to cancer cell growth and metabolism (Abedini, et al., 2025). Terms such as “lipid and atherosclerosis” and “positive regulation of glycolytic process” highlight the metabolic reprogramming common to both diseases, consistent with altered glucose and lipid metabolism in DM and the Warburg effect in HNSCC (Zou, et al., 2025). Notably, “insulin receptor binding” (GO: 0005158) provides a direct mechanistic bridge, supporting the hypothesis that hyperinsulinemia and insulin resistance may contribute to tumorigenesis via PI3K/AKT or MAPK pathways (Caruso, et al., 2025). Moreover, immune-related enrichment such as “Pathogenic *E. coli* infection” suggests shared inflammatory responses and potential microbiome dysbiosis. The recurrence of genes like APP, SRC, and CASP3 across multiple functional categories underscores their centrality in mediating these overlapping biological processes, positioning them as key candidates in the shared pathophysiology of DM and HNSCC.

### 4.5 Strengths and limitations

This study integrates clinical modeling with literature-driven bioinformatics, offering a multidimensional view of the DM-HNSCC association. The simulation-based co-occurrence modeling, sex-stratified analysis, and gene-pathway network construction strengthen the reliability and interpretability of findings.

However, several limitations should be acknowledged. First, while our co-occurrence modeling was based on real-world data, it partially relies on simulated population estimates, which may not fully capture real-world complexity. Second, the gene overlap analysis was derived from literature-based data mining and may be subject to publication bias, potentially favoring well-studied genes. Although we applied FDR correction and the Adjusted Binomial Method to improve reliability, bias cannot be entirely ruled out. Third, our functional findings are based on computational inference without direct experimental validation. The proposed pathways should therefore be viewed as hypothesis-generating. Future studies should validate key genes—such as CYP2C19, NLRP3, PVT1, and APP—using transcriptomic or proteomic data and functional assays. Lastly, the strong male predominance in our cohort (692 out of 728 patients) limits the robustness of sex-specific comparisons, especially in females. Further validation in larger, more balanced, and diverse cohorts is needed to confirm and extend these findings.

## 5 Conclusion

This study provides compelling evidence for a significant clinical and molecular association between diabetes mellitus (DM) and head and neck squamous cell carcinoma (HNSCC). Using an integrative approach combining a large retrospective clinical cohort, simulation-based co-occurrence modeling, and bioinformatics analyses, we demonstrated that DM is associated with an elevated risk of HNSCC, particularly among males. At the molecular level, we identified a set of overlapping genes functionally enriched in inflammation, metabolism, and neurological signaling pathways—four of which (*CYP2C19*, *NLRP3*, *PVT1*, and *APP*) form a core regulatory network potentially mediating the impact of DM on HNSCC development.

These findings support the hypothesis that shared biological mechanisms underlie the comorbidity between DM and HNSCC. The identification of candidate genes and pathways provides a foundation for future experimental validation and the development of predictive biomarkers or therapeutic targets. Ultimately, this research contributes to a deeper understanding of the interplay between metabolic disorders and cancer and underscores the importance of interdisciplinary strategies in managing patients with complex chronic conditions.

## Data Availability

The original contributions presented in the study are included in the article/Supplementary Material, further inquiries can be directed to the corresponding authors.
